# Aldehyde Dehydrogenase 2 Protects Against Post-Cardiac Arrest Myocardial Dysfunction Through a Novel Mechanism of Suppressing Mitochondrial Reactive Oxygen Species Production

**DOI:** 10.3389/fphar.2020.00373

**Published:** 2020-03-27

**Authors:** Rui Zhang, Baoshan Liu, Xinhui Fan, Wenjun Wang, Tonghui Xu, Shujian Wei, Wen Zheng, Qiuhuan Yuan, Luyao Gao, Xinxin Yin, Boyuan Zheng, Chuanxin Zhang, Shuai Zhang, Kehui Yang, Mengyang Xue, Shuo Wang, Feng Xu, Jiali Wang, Yihai Cao, Yuguo Chen

**Affiliations:** ^1^Department of Emergency Medicine, Qilu Hospital, Shandong University, Jinan, China; ^2^Shandong Provincial Clinical Research Center for Emergency and Critical Care Medicine, Institute of Emergency and Critical Care Medicine of Shandong University, Qilu Hospital, Shandong University, Jinan, China; ^3^Key Laboratory of Emergency and Critical Care Medicine of Shandong Province, Key Laboratory of Cardiopulmonary-Cerebral Resuscitation Research of Shandong Province, Shandong Provincial Engineering Laboratory for Emergency and Critical Care Medicine, Qilu Hospital, Shandong University, Jinan, China; ^4^The Key Laboratory of Cardiovascular Remodeling and Function Research, Chinese Ministry of Education, Chinese Ministry of Health and Chinese Academy of Medical Sciences, The State and Shandong Province Joint Key Laboratory of Translational Cardiovascular Medicine, Qilu Hospital, Shandong University, Jinan, China; ^5^Department of Microbiology, Tumor and Cell Biology, Karolinska Institute, Stockholm, Sweden

**Keywords:** aldehyde dehydrogenase 2, cardiopulmonary resuscitation, post-cardiac arrest myocardial dysfunction, cardiomyocyte death, mitochondrial reactive oxygen species

## Abstract

Post-cardiac arrest myocardial dysfunction significantly contributes to early mortality after the return of spontaneous circulation. However, no effective therapy is available now. Aldehyde dehydrogenase 2 (ALDH2) enzyme has been shown to protect the heart from aldehyde toxicity such as 4-hydroxy-2-nonenal (4-HNE) and oxidative stress. In this study, we evaluated the effect of enhanced activity or expression of ALDH2 on post-cardiac arrest myocardial dysfunction and survival in a rat cardiac arrest model. Furthermore, we elucidated the underlying mechanisms with a focus on mitochondrial reactive oxygen species (ROS) production in a cell hypoxia/reoxygenation model. A total of 126 rats were used for the ALDH2 activation or cardiac overexpression of ALDH2 studies. Randomization was done 10 min before the respective agonist injection or *in vivo* gene delivery. We showed that enhanced activity or expression of ALDH2 significantly improved contractile function of the left ventricle and survival rate in rats subjected to cardiac arrest-cardiopulmonary resuscitation procedure. Moreover, ALDH2 prevented cardiac arrest-induced cardiomyocyte death from apoptosis and mitochondrial damage. Mechanistically, 4-HNE, a representative substrate of ALDH2, was dominantly increased in the hypoxia/reoxygenation-exposed cardiomyocytes. Direct addition of 4-HNE led to significantly augmented succinate accumulation and mitochondrial ROS production. Through metabolizing 4-HNE, ALDH2 significantly inhibited mitochondrial ROS production. Our findings provide compelling evidence of the cardioprotective effects of ALDH2 and therapeutic targeting this enzyme would provide an important approach for treating post-cardiac arrest myocardial dysfunction.

## Introduction

Sudden cardiac arrest remains a major public health burden in terms of high mortality and morbidity worldwide (Hayashi et al., 2015). While efforts to initiate rapid cardiopulmonary resuscitation (CPR), e.g., establishment of medical emergency outreach teams, have improved the return of spontaneous circulation (ROSC) to 30–40% of patients with cardiac arrest, the mortality thereafter remains >50% (Neumar et al., 2008; Hazinski et al., 2015). Post-cardiac arrest myocardial dysfunction is an important cause of circulatory failure and early mortality after ROSC (Kern et al., 1996; Stub et al., 2011). Among the multiple factors, ischemia/reperfusion injury plays an essential role in the pathological progression of myocardial dysfunction. However, there is currently no effective therapeutic approach for post-cardiac arrest myocardial dysfunction as well as ischemia/reperfusion injury.

Mitochondria, as the center of energy supply and reactive oxygen species (ROS) production, play a crucial role as targets and drivers of ischemia/reperfusion injury after cardiac arrest (Penna et al., 2013; Matsuura et al., 2017). One mitochondrial feature during the ischemia/reperfusion processes is the ROS production (Zweier et al., 1987; Murphy and Steenbergen, 2008; Chouchani et al., 2014). Recently, mitochondrial ROS have been shown to be an important factor in contributing sudden cardiac death and myocardial dysfunction (Dey et al., 2018). However, the mechanism of mitochondrial ROS production and mitochondria-targeted treatments designed to ameliorate mitochondrial oxidative stress are still under study (Murphy, 2016; Dey et al., 2018).

Aldehydes are generated through lipid peroxidation on mitochondrial and plasma membranes in response to oxidative stress and have been observed in the heart after cardiac arrest (Hayashida et al., 2012). Aldehydes can easily diffuse from the side of their origin (i.e., membranes) and reach and attack targets intracellularly and extracellularly, forming adducts with macromolecules including proteins, DNA, and lipids which usually modulates or disrupts their functions (Roede and Jones, 2010; Li et al., 2018). Aldehydes can impair mitochondria by attacking on Cys, Lys, or Arg amino acid residues of mitochondrial proteins, but the underlying mechanism is still unclear (Roede and Jones, 2010; Li et al., 2018). Therefore, clarifying the role of aldehydes in mitochondrial injury and effectively clearing these highly harmful aldehydes is crucial to protect mitochondria from ischemia/reperfusion injury and alleviate post-cardiac arrest myocardial dysfunction.

Aldehyde dehydrogenase 2 (ALDH2) has recently emerged as a critical health-promoting enzyme, which is primarily expressed in the mitochondria in a wide variety of organs, including the heart (Li et al., 2018). Previous studies have suggested that ALDH2 plays a central protective role in several types of cardiac diseases and global cellular oxidative stress mainly through metabolizing various aldehydes, such as 4-hydroxy-2-nonenal (4-HNE) which is the most abundant and reactive carbonyl species (Chen et al., 2014; Ji et al., 2016; Liu et al., 2018). However, the influence of ALDH2 on post-cardiac arrest myocardial dysfunction and mitochondrial ROS has not been investigated yet. Thus, the aims of this study are (1) to evaluate the effect of enhanced activity or expression of ALDH2 on myocardial dysfunction and survival after cardiac arrest, (2) to examine the importance of ALDH2 in reducing cardiomyocyte death and mitochondrial injury, and (3) to elucidate the underlying mechanisms by which ALDH2 exerts cardioprotection with a focus on mitochondrial ROS production.

## Methods

### Animals

**Figure 1:**
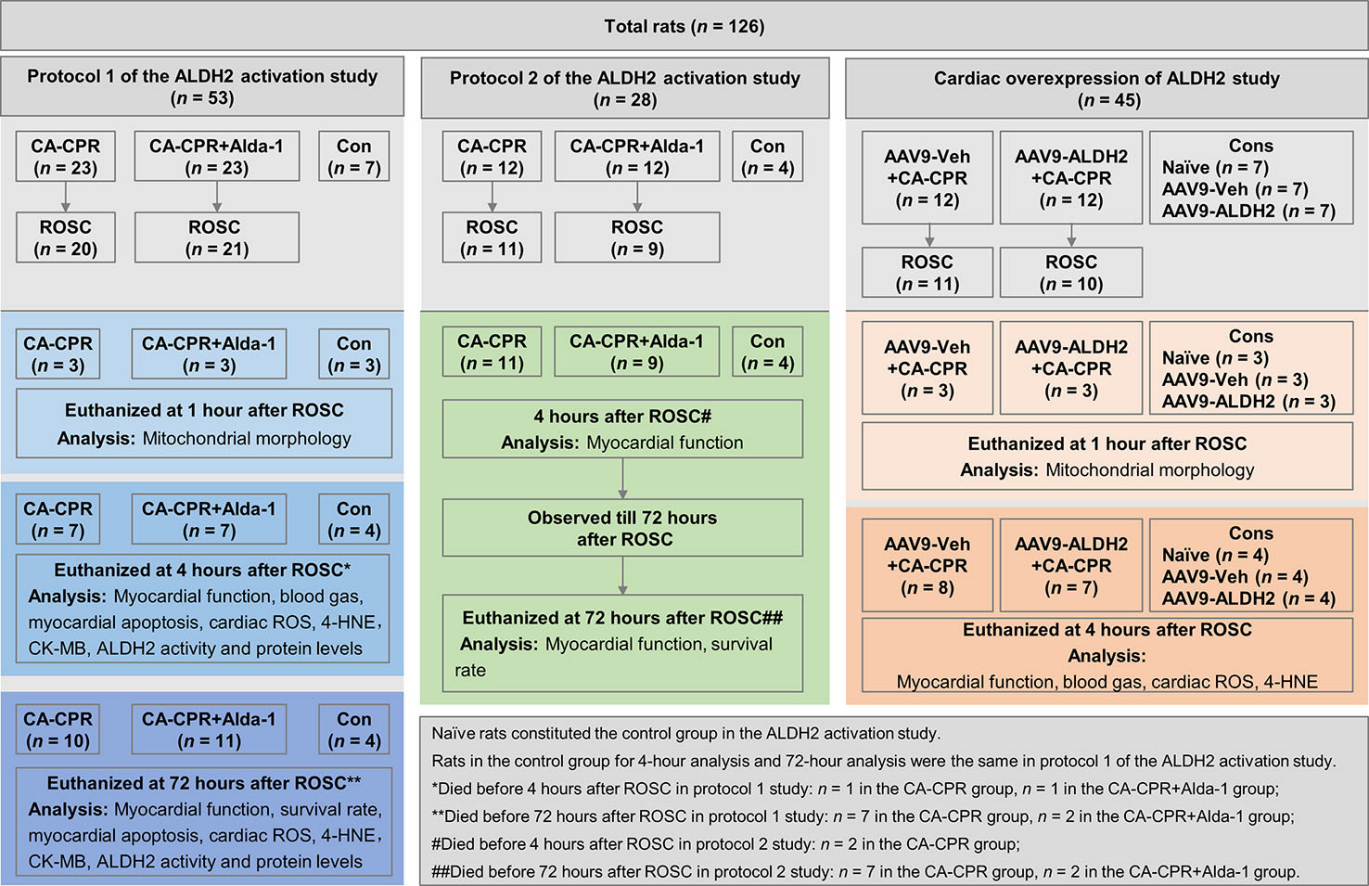
The flowchart of the animal study.

Adult male Wistar rats were purchased from the Department of Experimental Animals of Shandong University (Jinan, China) and acclimatized in the housing facility for at least 1 week before the cardiac arrest and CPR (CA-CPR) procedure. Rats were fed with normal chow and were free to access tap water at a constant temperature of 21.0°C ± 1.0°C, with a fixed 12-h light/dark cycle. A total of 126 rats were assigned to 1 of 3 animal study frameworks: (1) protocol 1 of the ALDH2 activation study (n = 53)—Alda-1 (10 mg/kg, Sigma-Aldrich, St. Louis, Missouri) was administered *via* intraperitoneal injection 30 min before cardiac arrest; (2) protocol 2 of the ALDH2 activation study (n = 28)— Alda-1 (10 mg/kg) was administered *via* intraperitoneal injection at the start of resuscitation; and (3) cardiac overexpression of ALDH2 study (n = 45)—adeno-associated virus (serotype 9) (AAV9)-ALDH2 or AAV9-Veh was delivered *via* tail vein injection at 2.5 × 10^11^ vector genomes/rat 4 weeks before cardiac arrest. In each animal cohort, rats were randomized respectively to CA-CPR group and CA-CPR+Alda-1 group, or AAV9-Veh+CA-CPR group and AAV9-ALDH2+CA-CPR group 10 min before the injection. Furthermore, rats in protocol 1 of the ALDH2 activation study were assigned to 1 of 3 tissue collection time points. At 1 h after ROSC, rats were euthanized for assessing mitochondrial morphology of heart. At 4 h after ROSC, rats were euthanized for myocardial functional and histological assessment. At 72 h after ROSC, rats were euthanized for assessing survival rate, myocardial function, and histology. In protocol 2 of the ALDH2 activation, rats with ROSC were followed up for 72 h for survival rate analysis. The myocardial function was detected within 4 h and at 72 h after ROSC. In the cardiac overexpression of ALDH2 study, rats were assigned to two tissue collection time points. At 1 h after ROSC, rats were euthanized for assessing mitochondrial morphology of heart. At 4 h after ROSC, rats were euthanized for myocardial functional and histological studies. Additional details about animal study can be found in the flowchart in **Figure 1**. The study was approved by the Institutional Animal Care and Use Committee of Shandong University, in accordance with National Institutes of Health Guidelines.

### Cardiac Arrest and CPR Procedure

CA-CPR procedure was performed in rats as previously described with minor modifications (Huang et al., 2008; Kim et al., 2016; Wang et al., 2016). Briefly, rats were anesthetized with pentobarbital sodium (45 mg/kg, intraperitoneal injection). The oral trachea intubation with a 14-G cannula was performed and ventilator settings included a tidal volume of 0.7 ml/100 g, a respiratory rate of 70 breaths/min and FiO2 of 21%. PE-50 tubes filled with heparinized saline were inserted into the right femoral artery for blood pressure monitoring and blood gas sampling, and into the right femoral vein for epinephrine administration. The Millar pressure-volume catheter (ADInstruments, Sydney, Australia) was inserted through the right carotid artery and advanced into the left ventricle, as appropriate. The rectal temperature was monitored and maintained at 37°C ± 0.5°C by a heating pad. Blood pressure, left ventricular pressure, and needle-probe electrocardiogram monitoring data were recorded with the PowerLab acquisition system (ADInstruments). CA was induced by asphyxia *via* turning off the ventilator and clamping the endotracheal tube. CA was defined as the femoral mean arterial pressure (MAP) < 30 mmHg. After 8 min of asphyxia, the mechanical ventilator was reinitiated. The epinephrine (2 μg/100 g, once every 3 min) was administered, and chest compression (200 beats/min) was performed, attempting for 10 min at most. ROSC was defined as the return of sinus rhythm with a MAP ≥60 mmHg lasting for at least 5 min.

### Cells and Hypoxia/Reoxygenation Procedure

Rat cardiomyoblasts cell line (H9c2) and primary cardiomyocytes isolated from adult male wild type (WT) mice and ALDH2 knockout (KO) mice were used. Cells were cultured normally in a humidified incubator with 95% air/5% CO_2_ at 37°C. Hypoxia/reoxygenation procedure was induced by exposing cells in a hypoxic workstation (H35, Don Whitley Scientific, Bingley, United Kingdom) containing 94% N_2_, 5% CO_2_, and 1% O_2_ at 37°C with serum-deprived media for 4 h and then culturing cells under normal conditions with complete media for another 2 h. Alda-1 (20 mmol/L) or Daidzin (60 mmol/L, Sigma-Aldrich) was added to the media 30 min before hypoxia/reoxygenation.

### *In Vivo* Gene Delivery

The recombinant AAV9 vector carrying rat *ALDH2* with a cardiac troponin T (cTNT) promoter and green fluorescent protein (GFP) (AAV9-ALDH2) (Hanbio Inc, Shanghai, China) or carrying only cTNT and GFP (AAV9-Veh) as a negative control was delivered to male rats *via* a bolus tail vein injection at 2.5 × 10^11^ vector genomes/rat. After 4 weeks, the expression of GFP in the liver, skeletal muscle, and heart tissue in rats receiving gene delivery was observed under a fluorescence microscope (Olympus Corporation, Tokyo, Japan), and the expression levels of ALDH2 were detected by western blot analysis.

### Measurement of Myocardial Function

Left ventricular cardiac output (CO) and ejection fraction (EF) were measured by Millar pressure-volume catheter (ADInstruments). Left ventricular end diastolic diameter (LVEDD) and left ventricular end systolic diameter (LVESD) were measured to calculate EF, fractional shortening (FS), left ventricular end diastolic volume (LVEDV), and left ventricular end systolic volume (LVESV) by echocardiography (VisualSonics, Toronto, Canada) as described previously (Wang et al., 2011).

### Tissue Collection and Processing at Pre-Specified Time Points

Rats were euthanized by transcardial perfusion with saline and then hearts were harvested rapidly. At 1 h after ROSC, the left ventricular tissue was cut into ~1 mm^3^ pieces and each piece was fixed with glutaraldehyde overnight for the transmission electron microscope (TEM) exanimation. At 4 h and 72 h after ROSC, the left ventricular tissue was washed in saline, snap frozen in liquid nitrogen and stored at −80°C for ALDH2 activity, ALDH2 expression, and cardiac ROS analysis; otherwise, the left ventricular tissue was fixed in 4% formaldehyde, embedded in paraffin, and cut into sections of 6 μm in thickness to assess apoptosis.

### Fluorescently-Labeled Alda-1

Alda-1 was combined with lissamine rhodamine B200, a red natural luciferin, by electrostatic adherence (Jieyi Biotech, Shanghai, China) and administered to rats *via* intraperitoneal injection. After 4 h, the left ventricular tissue was observed under a fluorescence microscope (Olympus Corporation).

### Measurement of Myocardial Apoptosis

Apoptosis was assessed by transferase mediated dUTP nick-end labeling (TUNEL) staining using ApopTag^®^ In Situ Apoptosis Detection Kits (Millipore, Burlington, Massachusetts) (Tan et al., 2012; Liu et al., 2017). After staining, the sections were observed under a fluorescence microscope (Olympus Corporation) and measured by Image-Pro Plus 6.0 (Media Cybernetics, Rockville, Maryland).

### Examination of Mitochondrial Morphology

Thin sections (70 nm) of left ventricular tissue were stained and examined using a scanning TEM (JEOL Ltd., Tokyo, Japan) (Huang et al., 2011). The severity of mitochondrial structural damage was semi-quantified using Flameng grading of 1 through 5 as described previously (Flameng et al., 1980; Hawong et al., 2015).

### Determination of Plasma Creatine Kinase-MB (CK-MB)

Blood samples were centrifuged at 1000 × g for 20 min to collect plasma. The levels of CK-MB were measured using enzyme-linked immunosorbent assay (ELISA) (Cloud-clone, Wuhan, China).

### Determination of Blood Gas

Arterial blood was obtained at 15 min, 1 h, and 4 h after ROSC and blood gas profiles (pH, PaO_2_, PaCO_2_, glucose, and lactate) were measured immediately using automated blood gas analyzer (Instrumentation Laboratory, Bedford, Massachusetts).

### Measurement of ALDH2 Activity

The mitochondria were isolated from myocardial tissue using the Tissue Mitochondria Isolation Kit (Beyotime, Nanjing, China). The mitochondria were sonicated, centrifuged at 11,000 × g for 10 min at 4°C and the supernatant was collected to measure ALDH2 activity. The supernatant was incubated with 50 mmol/L sodium pyrophosphate, 2.5 mmol/L NAD^+^, and 10 mmol/L propionaldehyde for 10 min. Acetaldehyde as the substrate of ALDH2 was oxidized to acetic acid, whereas NAD^+^ was reduced to NADH which was used to determine ALDH2 activity. Production of NADH was determined by spectrophotometric absorbance at 340 nm (Thermo Scientific, Waltham, Massachusetts) (Wang et al., 2011; Tan et al., 2012).

### Isolation of Primary Cardiomyocytes From Adult Mice

The ALDH2 KO mice were provided by University of Occupational and Environmental Health (Fukuoka, Japan). The cardiomyocytes were isolated from adult male WT mice and ALDH2 KO mice (all were C57BL/6 background) as described previously (Ackers-Johnson et al., 2016; Pang et al., 2016). Briefly, mice were sacrificed after anaesthetized with 2% isoflurane, and hearts were rapidly excised and mounted onto a temperature-controlled (37°C) Langendorff system (ADInstruments). The hearts were perfused retrogradely through the aorta with collagenase II (Sigma-Aldrich), collagenase IV (Sigma-Aldrich), and collagenase IV (Sigma-Aldrich) for about 20 min until digestion was apparent. The digested left ventricles were then cut into ~1 mm^3^ pieces and dissociated by pipetting 2 min. After four sequential rounds of gravity settling using three intermediate calcium reintroduction buffers to gradually restore calcium concentration to physiological levels, the cell pellet which was enriched with myocytes was collected and used for the experiments within 8 h after isolation.

### Measurement of Mitochondrial ROS, Cellular ROS, and Cardiac ROS

The mitochondrial ROS levels in H9c2 cells and primary cardiomyocytes were measured with MitoSOX Red reagent, a mitochondrial superoxide (O_2_^-^) indicator (Invitrogen, Carlsbad, California) (Roberge et al., 2014; Ji et al., 2016). Cells were incubated with 5 μmol/L MitoSOX Red for 10 min at 37°C protected from light. The cellular ROS levels in H9c2 cells were measured by incubating with 10 μmol/L 2′,7′-dichlorofluorescin diacetate (DCFH-DA) (Beyotime) for 30 min at 37°C protected from light which was converted to fluorescent 2′,7′- dichlorofluorescein (DCF) by ROS (Ji et al., 2016). The O_2_^-^ levels in cardiac tissue were measured using dihydroethidium (DHE) (Beyotime), an oxidative fluorescent dye (Ji et al., 2016; Zhang et al., 2016). Frozen sections (6 μm) of myocardium were incubated with 10 μmol/L DHE for 30 min at 37°C protected from light and incubated with DAPI (Boster, Wuhan, China) for 2 min to label nuclei. After incubation, cells and tissue sections were observed under a fluorescence microscope (Olympus Corporation). The intensity of fluorescence was quantified by ImageJ software (U.S. National Institutes of Health, Bethesda, Maryland). Three samples of each group were studied and five randomly selected fields of each sample were evaluated. The mean intensity of fluorescence of the five fields for each sample was used for statistical analysis.

### Measurement of Mitochondrial Respiratory Function

Mitochondrial respiratory function evaluated by oxygen consumption rate (OCR) was assessed using a Seahorse XF^e^24 analyzer with the Seahorse XF Cell Mito Stress Test Kit (Agilent Technologies, Santa Clara, California) as described previously (Ikeda et al., 2016; Tsushima et al., 2018). Briefly, ~10^5^ cells were seeded in a 24-well plate specific for the Seahorse XF^e^24 instrument and underwent hypoxia/reoxygenation procedure. After the hypoxia/reoxygenation procedure, cells were equilibrated with XF assay media supplemented with 25 mmol/L glucose, 1 mmol/L sodium pyruvate, and 2 mmol/L L-glutamine. Then, the OCR was analyzed by Seahorse XF^e^24 analyzer with the following inhibitors: 1 μmol/L oligomycin (Oligo), 1 μmol/L carbonyl cyanide 4-(trifluoromethoxy) phenylhydrazone (FCCP), and 0.5 μmol/L rotenone/antimycin A (Rot/Ant).

### Adenosine 5'-Triphosphate (ATP) Assay

Cellular ATP contents were measured by a firefly luciferase-based ATP assay kit (Beyotime) according to the manufacturer’s instructions. Briefly, cells were schizolysized and centrifuged at 12,000 × g for 5 min at 4°C to collect the cell supernatant which was then mixed with ATP detection working solution. The protein concentration of sample was measured and the standard curve of ATP concentration was prepared. The ATP level was measured based on the emitted light by a luminescence plate reader (Thermo Scientific).

### Measurement of Succinate Levels

The levels of succinate were assessed colorimetrically using the established Succinate Assay Kit (Abcam, Cambridge, UK) according to the manufacturer’s instructions (Karlstaedt et al., 2016). Briefly, cells were washed with cold PBS, resuspended in succinate assay buffer, homogenized, and centrifuged for 12,000 × g for 5 min at 4°C to collect supernatant. The samples were then incubated with the reaction mix for 30 min at 37°C. The standard curve of succinate concentration was prepared. The level of succinate was measured colorimetrically at 450nm (Thermo Scientific).

### Measurement of Mitochondrial Membrane Potential

The mitochondrial membrane potential was measured by incubating cells with 100 nmol/L TMRM (Invitrogen) for 30 min at 37°C. After rinsed twice, cells were observed under a fluorescence microscope (Olympus Corporation). The intensity of fluorescence was quantified by ImageJ software (U.S. National Institutes of Health) and was used to indicate the mitochondrial membrane potential. Three samples of each group were studied and five randomly selected fields of each sample were evaluated. The mean value of the five fields for each sample was used for statistical analysis.

### Assessment of Succinate Dehydrogenase (SDH) Carbonylation

To detect the levels of SDH carbonylation, SDH was immunoprecipitated by incubating 500 μg cell lysate with 2–10 μg anti-SDHA antibody (Abcam) overnight at 4°C, followed by incubation with 20 μl protein A/G agarose (Beyotime) for 2 h at 4°C. Immunoprecipitates were washed, resuspended in 1× sample buffer, boiled for 5 min, and analyzed by western blot analysis.

### Cell Treatment With 4-HNE

When detecting the level of mitochondrial ROS, cellular succinate, and mitochondrial membrane potential, 4-HNE (BioVision, Milpitas, California) with different concentrations (10 μM, 20 μM, or 40 μM) was incubated with ~10^6^ cells for 4 h. And when detecting SDH carbonylation, 40 μM 4-HNE was incubated with ~5×10^6^ cells for 4 h.

### Western Blot Analysis

Protein samples were separated by SDS-PAGE and transferred to nitrocellulose membranes (Millipore). After blocked with 5% milk, the blots were probed with antibodies against ALDH2 (1:1,000, Abcam), β-actin (1:1,000, Cell Signaling Technology, Danvers, Massachusetts), and 4-HNE-protein adducts (1:1,000, Abcam). For assessment of SDH carbonylation, blots were probed with anti-DNPH antibody (1:1,000, Abcam) after incubated with 0.5 mmol/L 2, 4-dinitro phenylhydrazine (DNPH, Sigma-Aldrich) for 30 min or probed with anti-SDHA antibody (1:1,000, Abcam) (Wu et al., 2016; Li et al., 2018). Membranes were washed and incubated with horseradish peroxidase-conjugated secondary antibodies (1:10,000) for 2 h and detected using the chemiluminescence method. The intensity of the bands was quantified by ImageJ software (U.S. National Institutes of Health).

### Immunofluorescence Staining

The distribution of 4-HNE-protein adducts in cells was detected using immunofluorescence staining. Briefly, cells grown on cover slip were fixed with 4% formaldehyde and permeated with 0.5% Triton X-100. After blocked with goat serum, cells were incubated with primary antibody against 4-HNE-protein adducts (1:1,000, Abcam) overnight. Cells were washed and incubated with secondary antibody for 1 h. Then, cells were incubated with 100 nmol/L Mito-tracker Red (Beyotime) for 30 min at 37°C protected from light and incubated with DAPI (Boster) for 2 min to label nuclei. The fluorescence of 4-HNE-protein adducts, Mito-tracker Red and DAPI was observed under a fluorescence microscope (Olympus Corporation).

### Statistical Analysis

The continuous data were presented as mean ± SEM. Group comparisons were performed by one-way analysis of variances (ANOVA) with Tukey’s *post hoc* test or Student’s t-test. Comparisons between time-based measurements within each group were performed by repeated-measures ANOVA. Survival was presented by Kaplan-Meier curves, and the log-rank test was used for comparing survival rate between groups. A *P* value <0.05 was considered statistically significant (2-tailed).

## Results

### Baseline and Procedural Characteristics of the Animal Study

There were no significant differences in the baseline body weight, heart rate, MAP, left ventricular CO, EF or FS between the CA-CPR group and the CA-CPR+Alda-1 group in the ALDH2 activation study (**Supplemental Table 1**). In addition, no differences were observed in cardiac arrest duration, CPR duration, or ROSC rate between the CA-CPR group and the CA-CPR+Alda-1 group in the ALDH2 activation study (**Supplemental Table 2**). Similar results with regard to the baseline and procedural features were found in the cardiac overexpression of ALDH2 study (**Supplemental Tables 3** and **4**).

### Activation of ALDH2 Effectively Improves Post-Cardiac Arrest Myocardial Dysfunction and Survival Rate

**Figure 2:**
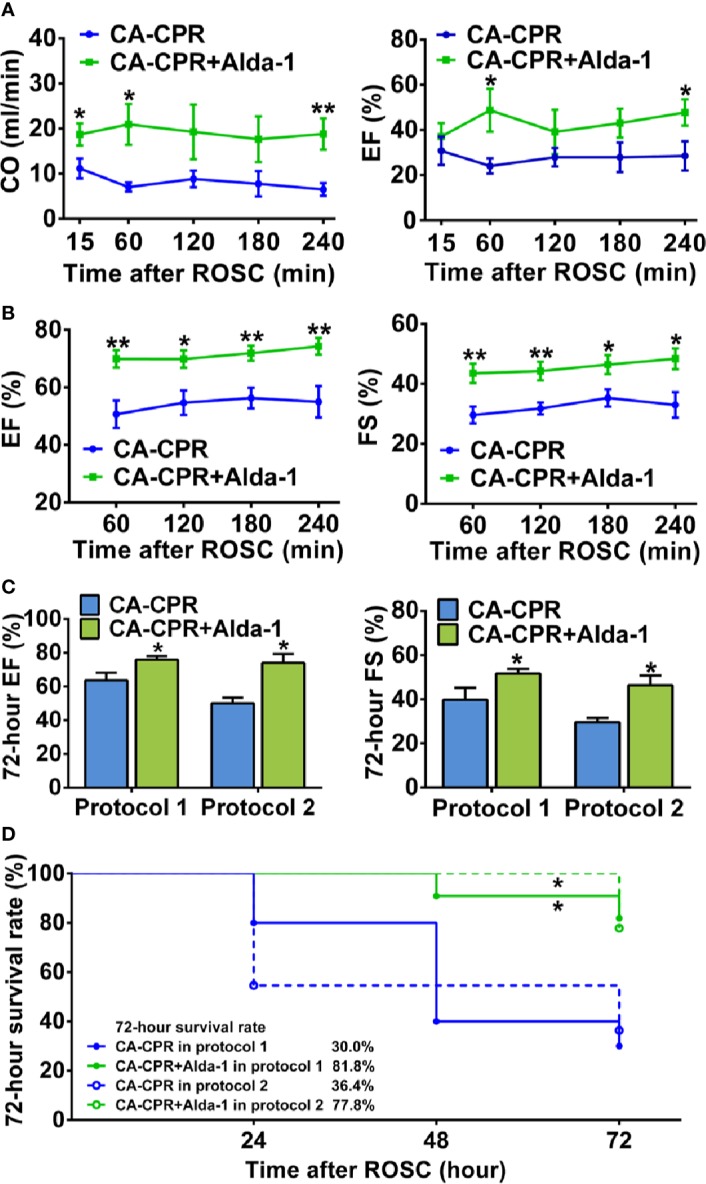
The effect of ALDH2 activation by Alda-1 on post-cardiac arrest myocardial dysfunction and survival rate. **(A)** Left ventricular cardiac output (CO) and ejection fraction (EF) evaluated by Millar pressure-volume catheter within 4 h after ROSC in protocol 1 of the ALDH2 activation study (*n* = 6 animals per group). **(B)** Left ventricular EF and fractional shortening (FS) evaluated by echocardiography within 4 h after ROSC in protocol 2 of the ALDH2 activation study (*n* = 9 animals per group). **(C)** Left ventricular EF and FS evaluated by echocardiography at 72 h after ROSC in the two protocols of the ALDH2 activation study (*n* = 3–9 animals per group). **(D)** Kaplan-Meier survival curves within 72 h after ROSC in the two protocols of the ALDH2 activation study. Myocardial function between groups was compared by Student’s t-test and time-based measurements within each group were compared by repeated-measures ANOVA. The survival rate between groups was compared by the log-rank test. Data are presented as mean ± SEM. *P < 0.05, **P < 0.01 versus CA-CPR group.

Myocardial function within 4 h after ROSC, as evaluated by left ventricular CO and EF using Millar pressure-volume catheter, was markedly reduced in all rats subjected to CA-CPR procedure (**Figure 2A**) compared with their respective baseline in protocol 1 of the ALDH2 activation study (**Supplemental Table 1**). Alda-1 reached the heart after intraperitoneal administration (**Supplemental Figure 1**) and significantly increased both left ventricular CO and EF, with the improvement in left ventricular CO occurring as early as 15 min after ROSC and the improvement in left ventricular EF occurring at 1 h after ROSC (**Figure 2A**). Similar trends for 4-h myocardial function after ROSC, as evaluated by left ventricular EF and FS using echocardiography, were observed in protocol 2 of the ALDH2 activation study (**Figure 2B**). Taken together, these results demonstrate that upregulation of ALDH2 activity by Alda-1 improves myocardial function within 4 h after ROSC.

We further evaluated left ventricular EF and FS at 72 h after ROSC using echocardiography in the CA-CPR group and the CA-CPR+Alda-1 group in the two protocols of the ALDH2 activation study. The impairment of left ventricular EF and FS remained significant in the CA-CPR group compared with their respective baseline in both protocol 1 and protocol 2; however, left ventricular EF and FS in the CA-CPR+Alda-1 group were restored to 92.0 and 89.7% of their baseline levels, respectively, in protocol 1 and restored to 86.3 and 82.5% of their baseline levels, respectively, in protocol 2 (*P* > 0.05 versus their respective baselines, **Figure 2C** and **Supplemental Table 1**). The 72-h survival rate was 30.0% in the CA-CPR group, whereas it was 81.8% in the CA-CPR+Alda-1 group in protocol 1. The log-rank test revealed markedly improved survival rate in the CA-CPR+Alda-1 group compared with the CA-CPR group (*P* < 0.05, **Figure 2D**). The same trend for survival rate between the CA-CPR group and CA-CPR+Alda-1 group was observed in protocol 2 (**Figure 2D**). Collectively, these results demonstrate that upregulation of ALDH2 activity reveals significant improvement in the 72-h myocardial function and survival rate.

We also determined the activity and expression levels of ALDH2 in myocardium in rats subjected to CA-CPR procedure. The results showed that the activity of ALDH2 was reduced by 58.8% at 4 h and 64.8% at 72 h after ROSC, respectively, which was significantly elevated by Alda-1; however, the expression levels of ALDH2 did not alter during the observation periods (**Supplemental Figure 2**). These findings can explain why upregulation of ALDH2 activity has a protective effect on post-cardiac arrest myocardial dysfunction.

### Activation of ALDH2 Attenuates Cardiac Arrest-Induced Cardiomyocyte Death and Mitochondrial Injury

**Figure 3:**
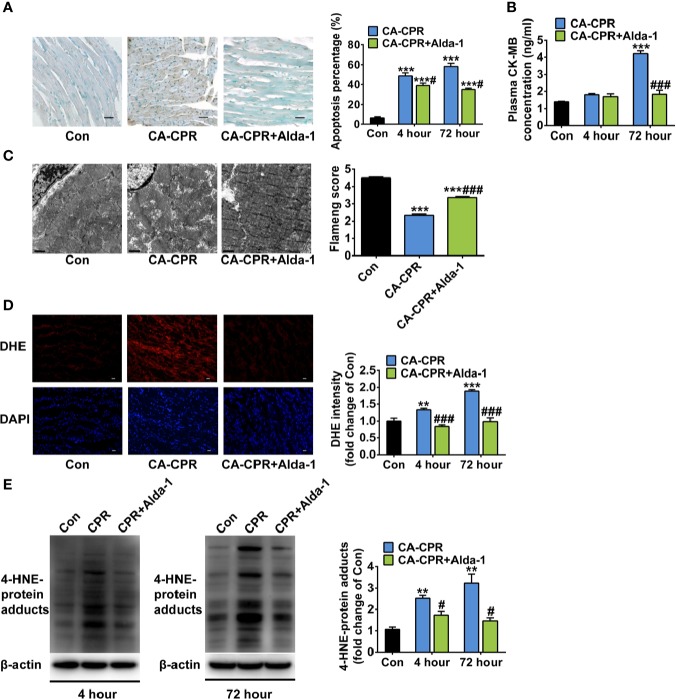
The effect of ALDH2 activation by Alda-1 on post-cardiac arrest cardiomyocyte death, mitochondrial structural damage, and cardiac ROS levels. **(A)** Representative photographs of cardiomyocyte apoptosis by TUNEL staining at 4 h after ROSC and quantification at 4 h and 72 h after ROSC (*n* = 3–4 animals per group). Scale bar = 100 μm. **(B)** The plasma levels of CK-MB at 4 h and 72 h after ROSC (*n* = 3–4 animals per group). **(C)** Representative photographs and quantification of mitochondrial morphology by TEM examination at 1 h after ROSC (*n* = 3 animals per group). Scale bar = 1 μm. **(D)** Representative photographs of dihydroethidium (DHE) staining at 4 h after ROSC and quantification at 4 h and 72 h after ROSC (n = 3–4 animals per group). Scale bar = 100 μm. **(E)** Representative immunoblots and quantification of 4-HNE-protein adducts at 4 h and 72 h after ROSC (*n* = 3–4 animals per group). Group comparisons were performed by ANOVA with Tukey’s *post hoc* test. Data are presented as mean ± SEM. **P < 0.01, ***P < 0.001 versus Con group; ^#^*P* < 0.05, ^###^*P* < 0.001 versus CA-CPR group.

**Figure 4:**
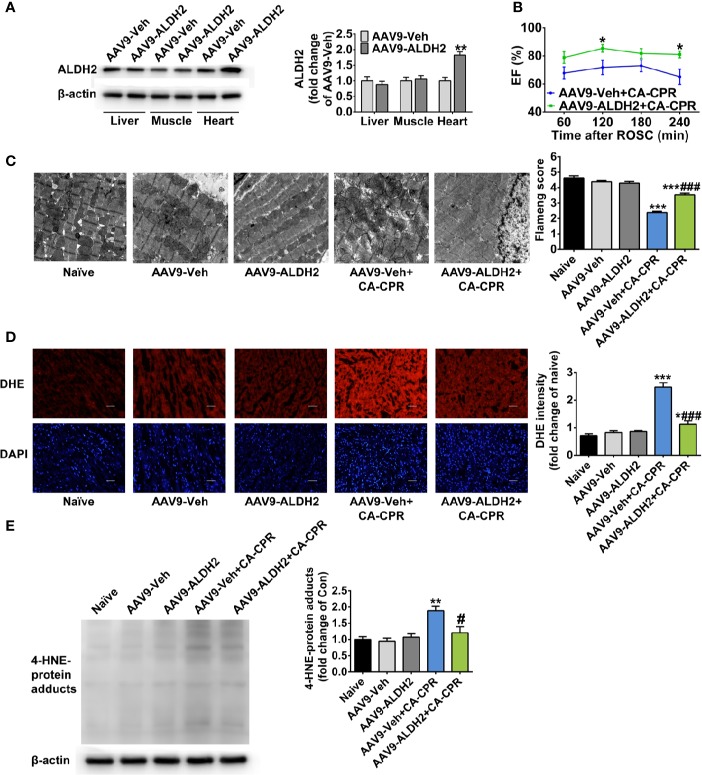
The effect of cardiac overexpression of ALDH2 on post-cardiac arrest myocardial dysfunction and mitochondrial structural damage. **(A)** Representative immunoblots and quantification of ALDH2 expression in the liver, skeletal muscle, and heart tissue (*n* = 5 animals per group). **P < 0.01 versus AAV9-Veh group. **(B)** Left ventricular EF within 4 h after ROSC in AAV9-Veh+CA-CPR and AAV9-ALDH2+CA-CPR group (*n* = 7–8 animals per group). *P < 0.05 versus AAV9-Veh+CA-CPR group. **(C)** Representative photographs and quantification of mitochondrial morphology by TEM examination at 1 h after ROSC (*n* = 3 animals per group). Scale bar = 1 μm. ***P < 0.001 versus Naïve group; ^###^*P* < 0.001 versus AAV9-Veh+CA-CPR group. **(D)** Representative photographs of dihydroethidium (DHE) staining at 4 h after ROSC (n = 4 animals per group). Scale bar = 100 μm. *P < 0.05, ***P < 0.001 versus Naïve group; ^###^*P* < 0.001 versus AAV9-Veh+CA-CPR group. **(E)** Representative immunoblots and quantification of 4-HNE-protein adducts at 4 h after ROSC (*n* = 4 animals per group). Myocardial function between groups was compared by Student’s t-test and time-based measurements within each group were compared by repeated-measures ANOVA. The mitochondrial morphology, DHE intensity, and 4-HNE-protein adducts between groups were compared by ANOVA with Tukey’s *post hoc* test. Data are presented as mean ± SEM. **P < 0.01 versus naïve group; ^#^*P* < 0.05 versus AAV9-Veh+CA-CPR group.

Because we saw no differences of the cardioprotection effect between these two protocols of ALDH2 activation study and in order to keep consistence with the ALDH2 overexpression study which enhanced ALDH2 activity before cardiac arrest, the rats in protocol 1 of the ALDH2 activation study were used for the following mitochondrial morphology and biomedical investigations. As expected, cardiac myocyte apoptosis was increased sharply at both 4 h and 72 h after ROSC in the CA-CPR group, which was significantly attenuated by Alda-1 (**Figure 3A**). The levels of plasma CK-MB were increased 3.0-fold at 72 h after ROSC, which were abolished by Alda-1 (**Figure 3B**). Consisted with these findings, obvious mitochondrial structural damage was observed at 1 h after ROSC in the CA-CPR group, which was partially reversed by Alda-1 (**Figure 3C**). The elevated cellular ROS levels (**Figure 3D**) and 4-HNE-protein adducts (**Figure 3E**) were also inhibited by Alda-1. However, there were no significant differences in the levels of blood gas (pH, PaO_2_, PaCO_2_, glucose, and lactate) between the CA-CPR group and the CA-CPR+Alda-1 group at 15 min, 1 h, or 4 h after ROSC, respectively (**Supplemental Table 5**). These results suggest that myocardial cell death and mitochondrial damage should be the disordered physiological processes that cause post-cardiac arrest myocardial dysfunction, whereas upregulation of ALDH2 activity could attenuate these disorders.

### Enhanced Expression of ALDH2 Improves Post-Cardiac Arrest Myocardial Dysfunction

To further validate the cardioprotective effect of ALDH2 activation, we examined whether enhancing the expression of ALDH2 had protective effect on post-cardiac arrest myocardial dysfunction. The AAV9 vector carrying ALDH2 with a cTNT promoter was constructed, which led to the increased expression of ALDH2 in the heart, but not in the liver or skeletal muscle (**Figure 4A** and **Supplemental Figure 3**). Cardiac specific overexpression of ALDH2 significantly improved left ventricular EF, evaluated by echocardiography, within 4 h after ROSC compared with that in the CA-CPR group (**Figure 4B**). The mitochondrial structural damage at 1 h after ROSC was significantly prevented by cardiac specific overexpression of ALDH2 (**Figure 4C**). The elevated cellular ROS levels (**Figure 4D**) and 4-HNE-protein adducts (**Figure 4E**) were also inhibited by cardiac specific overexpression of ALDH2. However, ALDH2 overexpression did not affect the levels of blood gas at 15 min, 1 h, or 4 h after ROSC (**Supplemental Table 6**). These findings indicate that enhancement of ALDH2 through both enzymatic activation and protein overexpression attenuates post-cardiac arrest myocardial dysfunction.

### Alda-1 Specifically Activates ALDH2 and Suppresses Hypoxia/Reoxygenation-Induced Mitochondrial ROS

**Figure 5:**
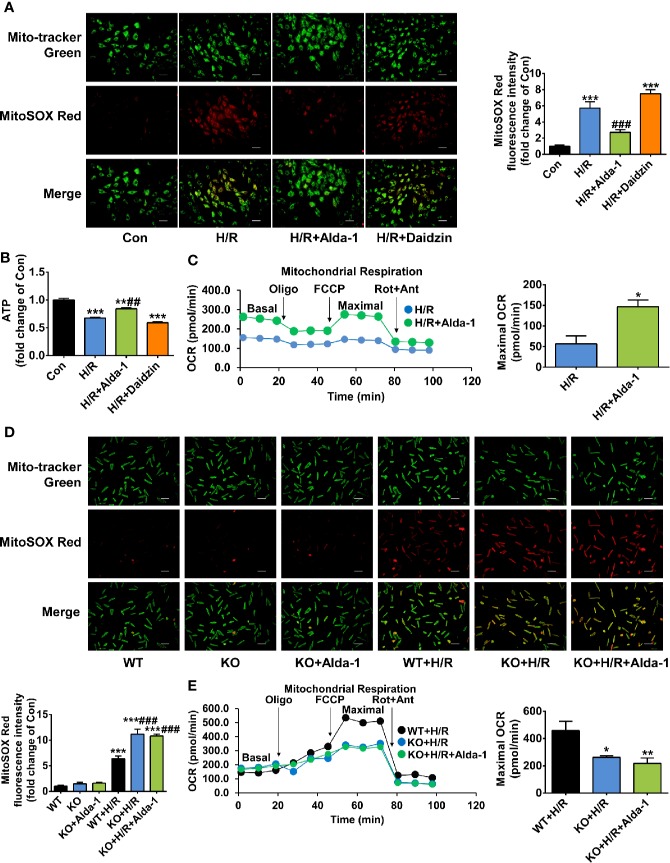
The effect of Alda-1 on mitochondrial ROS levels and mitochondrial energy generation capacity during hypoxia/reoxygenation (H/R). **(A–C)** Data are from H9c2 cells and are presented as mean ± SEM. **(A)** Representative photographs and quantification of mitochondrial ROS levels (*n* = 3 samples per group). Scale bar = 250 μm. ***P < 0.001 versus Con group; ^###^*P* < 0.001 versus H/R group. **(B)** The levels of cellular ATP (*n* = 3 samples per group). **P < 0.01, ***P < 0.001 versus Con group; ^##^*P* < 0.01 versus H/R group. **(C)** Mitochondrial respiratory function evaluated by OCR and quantification of maximal OCR (*n* = 3 samples per group). The maximal OCR was calculated by subtracting non-mitochondrial respiration rate (the final rate after Rot/Ant) from the maximal FCCP rate. *P < 0.05 versus H/R group. **(D, E)** Data are from primary cardiomyocytes isolated from adult male WT mice and ALDH2 KO mice, and are presented as mean ± SEM. **(D)** Representative photographs and quantification of mitochondrial ROS levels (*n* = 3 samples per group). Scale bar = 250 μm. ***P < 0.001 versus WT group; ^###^*P* < 0.001 versus WT+H/R group. **(E)** Mitochondrial respiratory function evaluated by OCR and quantification of maximal OCR (*n* = 5–6 samples per group). *P < 0.05, **P < 0.01 versus WT+H/R group. Group comparisons were performed by ANOVA with Tukey’s *post hoc* test or Student’s t-test.

We explored the molecular mechanisms by which ALDH2 exerted cardioprotection after cardiac arrest through regulation of its activity in a rat cardiomyoblast hypoxia/reoxygenation model, focusing on mitochondrial ROS. The levels of mitochondrial ROS were increased 5.7-fold during hypoxia/reoxygenation, the extent of which was significantly reduced by Alda-1; inhibition of ALDH2 by Daidzin did not further increase the mitochondrial ROS levels (**Figure 5A**). Similar inverse trends were observed for the cellular ATP levels (**Figure 5B**). The overall cellular ROS levels were also elevated during hypoxia/reoxygenation, which were inhibited by Alda-1 (**Supplemental Figure 4**). Additionally, mitochondrial respiratory dysfunction reflected by the reduced OCR under hypoxia/reoxygenation was markedly reversed by Alda-1 (**Figure 5C**). These results show the dramatically increased levels of mitochondrial ROS and the concomitant disorders of mitochondrial energy generation during reoxygenation could be inhibited by Alda-1, suggesting control of mitochondrial ROS should be an important function of ALDH2.

Furthermore, we assessed the specificity of Alda-1 on ALDH2, which has not been proved in the previous studies yet. Primary cardiomyocytes were obtained from ALDH2 KO mice and its background WT mice, respectively. ALDH2 KO cardiomyocytes generated more mitochondrial ROS under hypoxia/reoxygenation compared with the cardiomyocytes from WT mice; however, Alda-1 had no effect on the levels of mitochondrial ROS in ALDH2 KO cardiomyocytes (**Figure 5D**). Similar findings were observed for hypoxia/reoxygenation-induced mitochondrial respiratory dysfunction (**Figure 5E**). These results indicate that Alda-1 has no other targets in mitochondrial protection except interacting with ALDH2.

### 4-HNE Is Increased Under Hypoxia/Reoxygenation and Promotes Mitochondrial ROS Production

**Figure 6:**
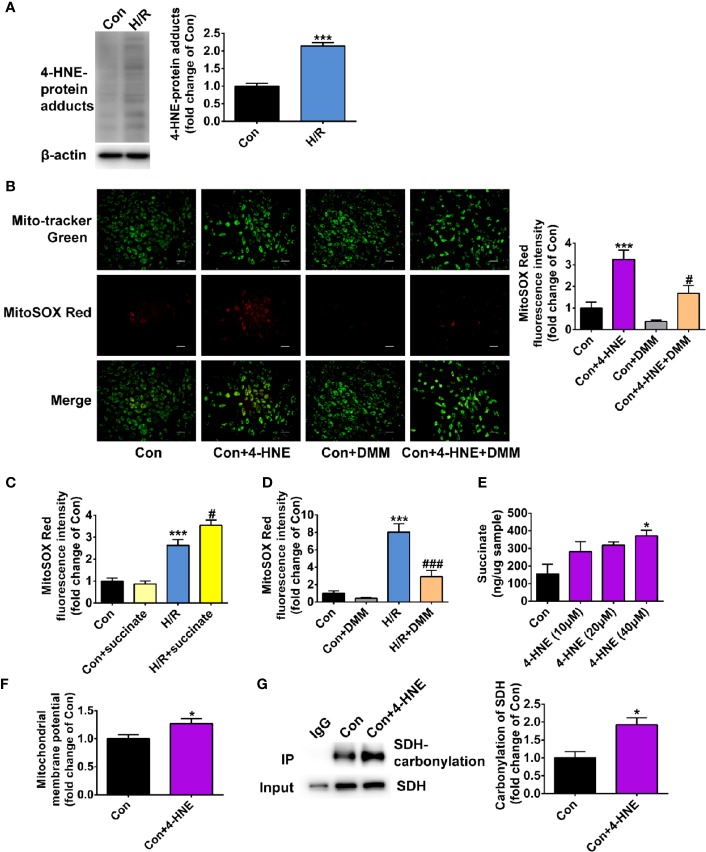
The effect of 4-HNE on mitochondrial ROS levels, succinate accumulation, mitochondrial membrane potential and SDH carbonylation. **(A)** Representative immunoblots and quantification of 4-HNE-protein adducts under hypoxia/reoxygenation (H/R) (*n* = 3 samples per group). **(B)** Representative photographs and quantification of mitochondrial ROS levels under the treatment of 4-HNE (40 μM) and 4-HNE (40 μM) +dimethyl malonate (DMM) (*n* = 3 samples per group). **(C, D)** The mitochondrial ROS levels after the addition of succinate or dimethyl malonate (DMM) during hypoxia/reoxygenation (H/R) (n = 4 samples per group). **(E)** The levels of succinate under the treatment of 4-HNE (*n* = 3 samples per group). **(F)** The quantification of mitochondrial membrane potential under the treatment of 4-HNE (40 μM) (*n* = 3 samples per group). **(G)** Representative immunoblots and quantification of SDH carbonylation under the treatment of 4-HNE (40 μM) (*n* = 3 samples per group). Group comparisons were performed by ANOVA with Tukey’s *post hoc* test or Student’s t-test. Data are presented as mean ± SEM. *P < 0.05, ***P < 0.001 versus Con group; ^#^*P* < 0.05 versus Con+4-HNE group or H/R group; ^###^*P* < 0.001 versus H/R group.

**Figure 7:**
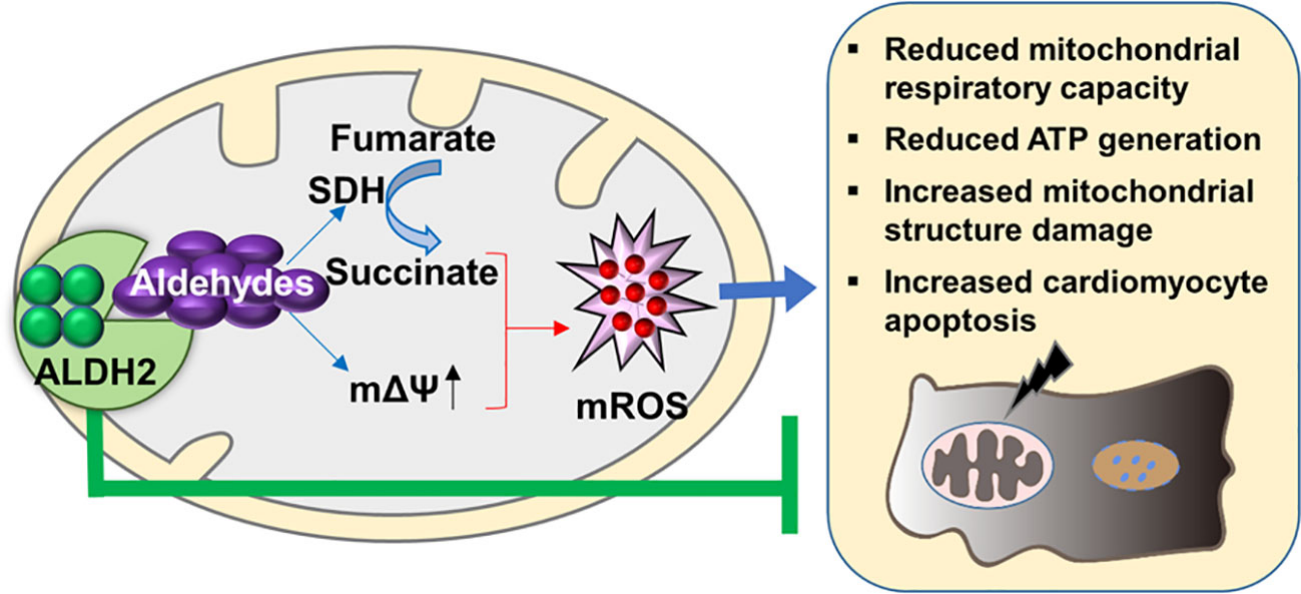
The proposed molecular mechanisms of the protective effect of ALDH2 on post-cardiac arrest myocardial dysfunction. The aldehydes, e.g. 4-HNE, which are accumulated during ischemia/reperfusion result in mitochondrial ROS (mROS) burst. The latter triggers a series of orchestrated events including reduced mitochondrial respiratory capacity, reduced ATP generation, increased mitochondrial structure damage, and cardiomyocyte death, thereby contributing to myocardial dysfunction. Evidence from this study show that targeting ALDH2 would provide an important approach for treating post-cardiac arrest myocardial dysfunction through inhibiting mitochondrial ROS production.

We further examined whether the effect of ALDH2 on inhibiting mitochondria ROS production during hypoxia/reoxygenation was attributed to its enzymatic function against toxic aldehyde overload. 4-HNE, a representative substrate of ALDH2, was selectively investigated. The 4-HNE-protein adducts were increased 2.1-fold after 4 h of hypoxia followed by 2 h of reoxygenation, which were distributed in both the mitochondria and the cytoplasm (**Figure 6A** and **Supplemental Figure 5**). Importantly, 4-HNE increased the levels of mitochondrial ROS 3.2-fold (**Figure 6B**). Since previous evidence has shown that the accumulated succinate is the main driver of mitochondrial ROS production (Chouchani et al., 2014), we hypothesized that 4-HNE may interfere with this process. We observed that the levels of mitochondrial ROS were aggravated by addition of succinate during hypoxia/reoxygenation (**Figure 6C**), which were blocked by dimethyl malonate (DMM), a membrane-permeable precursor of the SDH competitive inhibitor and SDH is a critical enzyme responsible for succinate generation (**Figure 6D**). It was interesting that 4-HNE stimulation resulted in the elevated succinate accumulation in a concentration-dependent manner (**Figure 6E**) and increased mitochondrial membrane potential (**Figure 6F**). Additionally, augmented SDH carbonylation was observed with 4-HNE treatment (**Figure 6G**). Treatment with DMM inhibited 4-HNE-induced increase of mitochondrial ROS levels (**Figure 6B**). Taken together, these data suggest that aldehydes play an essential role in mediating succinate accumulation and mitochondrial ROS production, which may explain why enhancement of ALDH2 inhibits mitochondrial ROS production during hypoxia/reoxygenation (**Figure 7**).

## Discussion

In this study, we demonstrate that enhanced activity or expression of ALDH2 attenuates myocardial dysfunction, cardiomyocyte death, and mitochondrial injury in a rat cardiac arrest model. Importantly, ALDH2 restores 72-h myocardial contractile function and substantially improves the survival rate after cardiac arrest. In the mechanistic studies, we reveal that aldehydes, 4-HNE, increased after ischemia/reperfusion injury which promotes succinate accumulation and mitochondrial ROS production. By clearing 4-HNE, ALDH2 suppresses mitochondrial ROS. Therefore, our results clearly suggest the protective role of ALDH2 in post-cardiac arrest myocardial dysfunction through a novel mechanism of suppressing aldehydes-mediating mitochondrial ROS production.

We used the asphyxia-induced cardiac arrest model in this study which is a well-established model to study cardiovascular dysfunction after cardiac arrest and the baseline cardiovascular parameters in this study are consistent with previous reports (Huang et al., 2007; Huang et al., 2008; Oh et al., 2017). We evaluated the effect of ALDH2 on post-cardiac arrest myocardial dysfunction through either upregulation of ALDH2 enzymatic activity or cardiac specific overexpression of ALDH2 in the animal studies. The cardioprotective performance of ALDH2 was established through these complete evaluations. Therefore, the beneficial role of ALDH2 in heart diseases is extended to the life-threatening cardiac arrest thus far. In addition, the unifying characteristics of these strategies are that the interventions were applied before the restoration of blood flow to the myocardium in rats subjected to cardiac arrest (Eltzschig and Eckle, 2011; Patil et al., 2015). While pharmacologic interventions before cardiac arrest are impossible due to the unexpected occurrence of cardiac events in routine settings, our results offer an original concept that therapies targeting ALDH2 in treating cardiac arrest could be initiated at an early time window, e.g., during basic life support. It is notable that although the mechanisms by which Alda-1 increases ALDH2 activity have been clarified in the previous literature (Perez-Miller et al., 2010), we first showed the specificity of Alda-1 on ALDH2, especially in mitochondrial protection. Our results suggest it is worth the effort to translate Alda-1 into clinical therapies or to explore new candidates which can enhance ALDH2 activity.

There are several other potentially therapeutic approaches that have been shown to be effective in improving post-cardiac arrest myocardial dysfunction. Pharmacological inhibition of mitochondrial permeability transition pore with cyclosporine A at the onset of resuscitation or after ROSC preserves myocardial function and attenuates post-cardiac arrest myocardial injury in the rabbit and rat models of cardiac arrest (Cour et al., 2011; Knapp et al., 2015). Natural hibernation signaling central mediator pentazocine, a δ-opioid receptor agonist, improves cardiac index after ROSC, which is returned to ~95% of baseline in a rat cardiac arrest model (Fang et al., 2006). In this study, we provide a new target for attenuating post-cardiac arrest myocardial dysfunction, which exhibits similar effects with previous studies. This evidence is all from animal studies; however, whether anyone could protect human victims of cardiac arrest requires further investigation.

We observed 4-HNE-overload and mitochondrial injury in heart after cardiac arrest. Aldehydes were found in heart and brain after cardiac arrest as reactive stress markers, however how aldehydes lead to cardiac injury and how to clear them were still indefinite (Hayashida et al., 2012; Liu et al., 2016). Mitochondrial damage in heart was observed from 1 min to 1 h after ROSC (Tsai et al., 2011; Huang et al., 2015). We found that mitochondrial ROS was involved in the mitochondrial dysfunction, impaired mitochondrial morphology, and cardiomyocytes apoptosis. Additionally, mitochondrial ROS were able to drive both acute emergent events, such as electrical instability responsible for sudden cardiac arrest, and chronic heart failure remodeling (Dey et al., 2018). However, protective treatments specifically targeted to mitochondria are still under study (Murphy, 2016; Dey et al., 2018). In this study, we found that ALDH2 could reduce mitochondrial ROS, therefore protecting mitochondria and heart after cardiac arrest.

Additionally, we provided a new understanding about the mechanisms of mitochondrial ROS production after cardiac arrest. In the present, there has no agreed conclusion about how mitochondrial ROS are produced. Some people believed that electron “leakage” from damaged mitochondrial electron transport chain during ischemia/reperfusion constituted a major source of mitochondrial ROS (Han et al., 2008). Recently, it was reported that there was a common pathway, that succinate oxidation and a high mitochondrial membrane potential were two essential requirements for mitochondrial ROS production (Chouchani et al., 2014; Chouchani et al., 2016; Mills et al., 2016). In this study, we found that 4-HNE mediate mitochondrial ROS production and amplify the ROS-induced ROS systems. 4-HNE is one of the lipid peroxidation-derived aldehydes which has been most intensively studied. We observed the 4-HNE could induce the carbonylation of SDH which is a critical enzyme responsible for succinate generation. Additionally, 4-HNE promoted succinate accumulation and led to increase in mitochondrial membrane potential and mitochondrial ROS levels. These findings provide novel evidence for the role of aldehydes in mitochondrial ROS production.

Several limitations should be considered. Firstly, although we identify the significant improvement of ALDH2 on post-cardiac arrest myocardial dysfunction and 72-h survival rate in a rat cardiac arrest model, we do not assess the long-term outcome. Secondly, we are unable to determine definitively whether the protective effect of ALDH2 is through suppressing mitochondrial ROS production during cardiac arrest due to the technique challenge for detecting mitochondrial ROS in living organisms as accurately as in cells. Thirdly, we did not test the dose-response study or more therapeutic windows within which enhancing ALD2 activity is beneficial after cardiac arrest. Thus, further investigations are needed to answer these questions.

In summary, we demonstrate that enhanced activity or expression of ALDH2 reduces cardiomyocyte death and mitochondrial injury, attenuates post-cardiac arrest myocardial dysfunction, and improves 72-h survival rate in a rat cardiac arrest model. Our results also imply that the protective role of ALDH2 may be through suppressing 4-HNE-mediating mitochondrial ROS production. These findings suggest therapeutic targeting ALDH2 would provide an important approach for treating post-cardiac arrest myocardial dysfunction and warrants clinical validation.

## Data Availability Statement

The datasets generated for this study are available on request to the corresponding authors.

## Ethics Statement

The animal study was reviewed and approved by the Institutional Animal Care and Use Committee of Shandong University.

## Author Contributions

YCh, YCa, JW, FX, RZ, BL, WW, SWe, QY, MX, and SWa designed research. RZ, BL, XF, WW, TX, WZ, LG, XY, BZ, CZ, SZ, and KY performed research. RZ, JW, BL, WW, and XF analyzed data. JW, YCh, YCa, RZ, BL, SWe, and QY wrote and revised the paper.

## Funding

This study was supported by the National Key Research and Development Program of China (2017YFC0908700, 2017YFC0908703), National Natural Science Foundation of China (81772036, 81671952, 81873950, 81873953, 81570401, 81571934), National Science and Technology Fundamental Resources Investigation Project (2018FY100600, 2018FY100602), Taishan Pandeng Scholar Program of Shandong Province (tspd20181220), Taishan Young Scholar Program of Shandong Province (tsqn20161065, tsqn201812129), Key Research and Development Program of Shandong Province (2018GSF118003), the Fundamental Research Funds of Shandong University (2018JC011), Science Foundation of Qilu Hospital of Shandong University (2015QLQN13), and the Natural Science Foundation of Shandong Province (ZR2014HP035).

## Conflict of Interest

The authors declare that the research was conducted in the absence of any commercial or financial relationships that could be construed as a potential conflict of interest.
