# Supplementary material for: Aldehyde Dehydrogenase 2 Protects Against Post-Cardiac Arrest Myocardial Dysfunction Through a Novel Mechanism of Suppressing Mitochondrial Reactive Oxygen Species Production
Source: Front Pharmacol. 2020 Mar 27;11:373. doi: 10.3389/fphar.2020.00373 (PMC7118728; doi:10.3389/fphar.2020.00373)
Supplement: Supplementary file 1 [file DataSheet_1.docx]

Supplementary Material

**Aldehyde dehydrogenase 2 protects against post-cardiac arrest myocardial dysfunction through a novel mechanism of suppressing mitochondrial reactive oxygen species production**

Rui Zhang, Baoshan Liu, Xinhui Fan, Wenjun Wang, Tonghui Xu, Shujian Wei, Wen Zheng, Qiuhuan Yuan, Luyao Gao, Xinxin Yin, Boyuan Zheng, Chuanxin Zhang, Shuai Zhang, Kehui Yang, Mengyang Xue, Shuo Wang, Feng Xu, Jiali Wang, Yihai Cao, Yuguo Chen

Corresponding authors:

Yuguo Chen ([chen919085@sdu.edu.cn](mailto:chen919085@sdu.edu.cn)), Yihai Cao (yihai.cao@ki.se) or Jiali Wang (wangjiali_2000@126.com)

**This PDF file includes:**

Supplemental Figure 1 to Supplemental Figure 5

Supplemental Table 1 to Supplemental Table 6

# Supplementary Figures

#
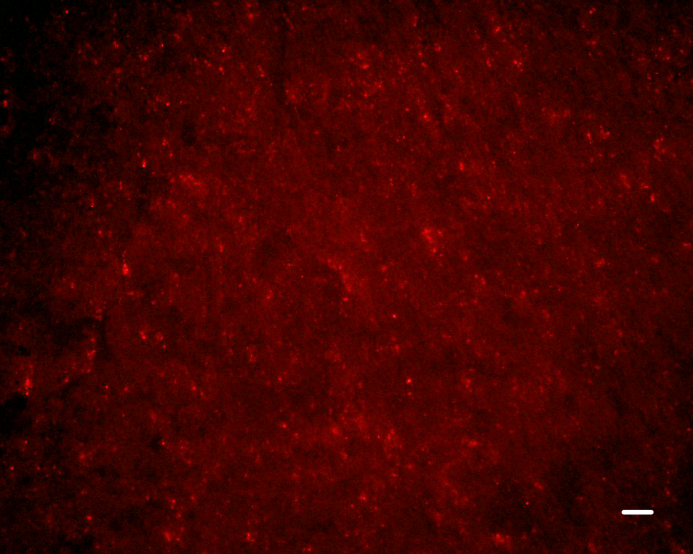


**Supplemental Figure 1.** The fluorescently-labeled Alda-1 in the heart tissue. The red fluorescence indicated that Alda-1 was distributed in the heart tissue after administration via intraperitoneal injection. Scale bar = 100 μm.

**
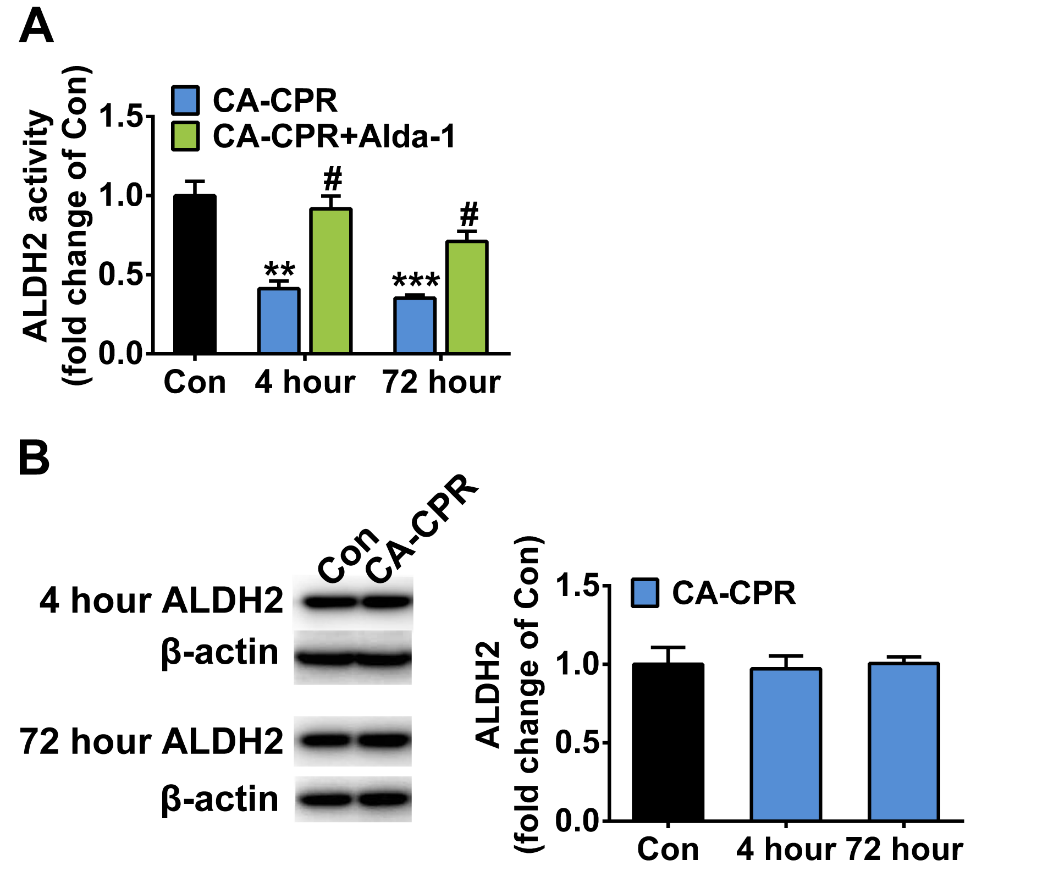
**

**Supplemental Figure 2.** The activity and expression levels of ALDH2 in myocardium in rats after cardiac arrest. (A) ALDH2 activity at 4 hours and 72 hours after ROSC (*n* = 3-5 animals per group). (B) Representative immunoblots and quantification of ALDH2 expression at 4 hours and 72 hours after ROSC (*n* = 3-6 animals per group). Data are presented as mean ± SEM. ***P* < 0.01, ****P* < 0.001 versus Con group; #*P* < 0.05 versus CA-CPR group.

**
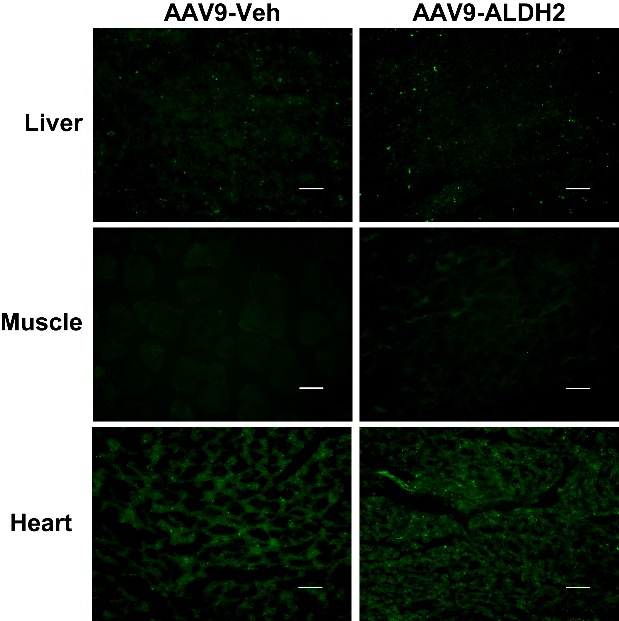
**

**Supplemental Figure 3.** The expression of GFP in the liver, skeletal muscle and heart tissue. The green fluorescence confirmed the heart tissue-specific gene expression of AAV9-Veh and AAV9-ALDH2 (both carrying GFP gene) in rats 4 weeks after receiving gene delivery. Scale bar = 500 μm.

**
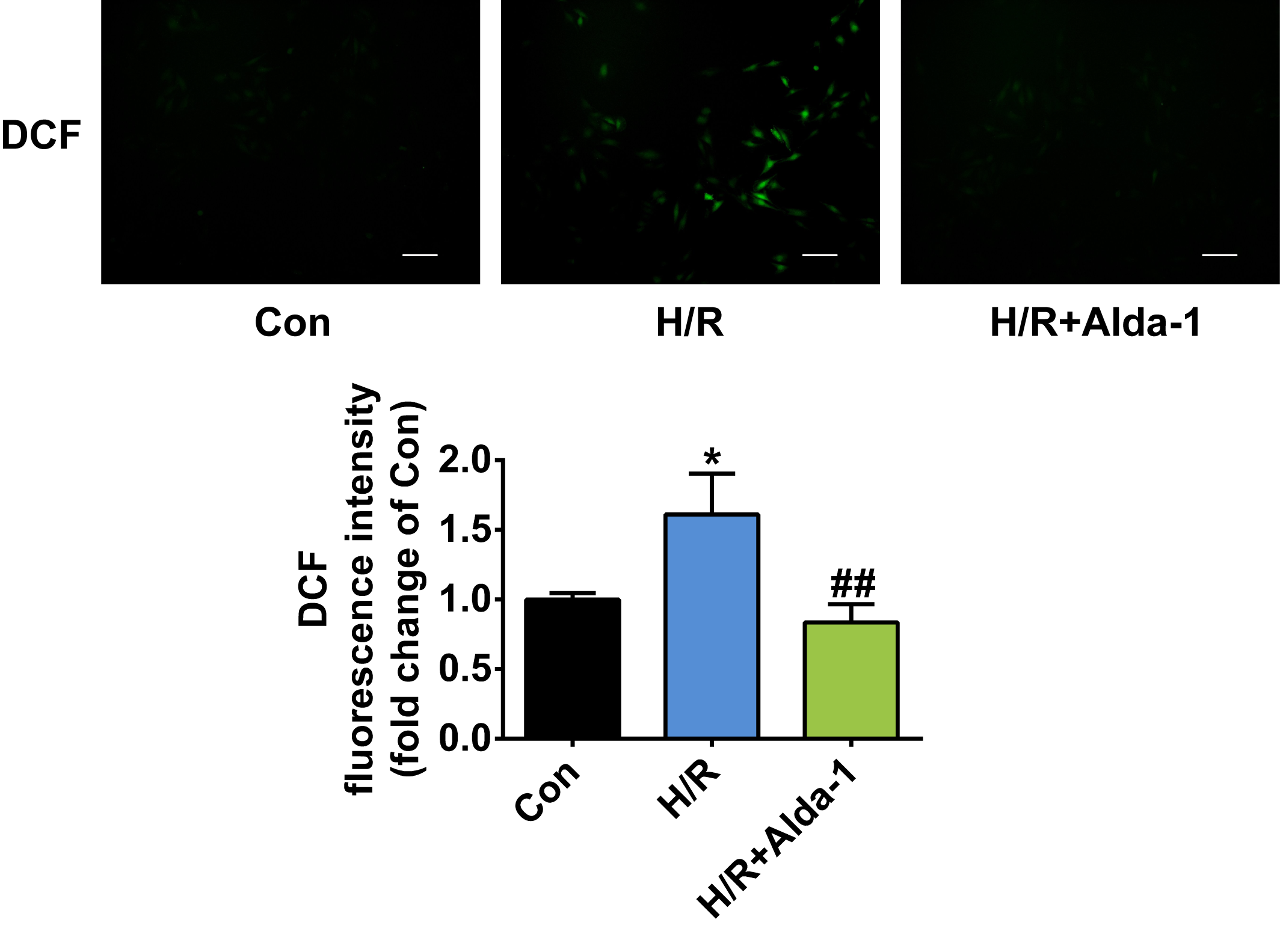
**

**Supplemental Figure 4.** Cellular ROS levels during hypoxia/reoxygenation. Representative photographs of cellular ROS and quantification during hypoxia/reoxygenation (H/R) and H/R+Alda-1 (*n* = 3 samples per group). Scale bar = 250 μm. Data are presented as mean ± SEM. **P* < 0.05 versus Con group; ##*P* < 0.01 versus H/R group.

**
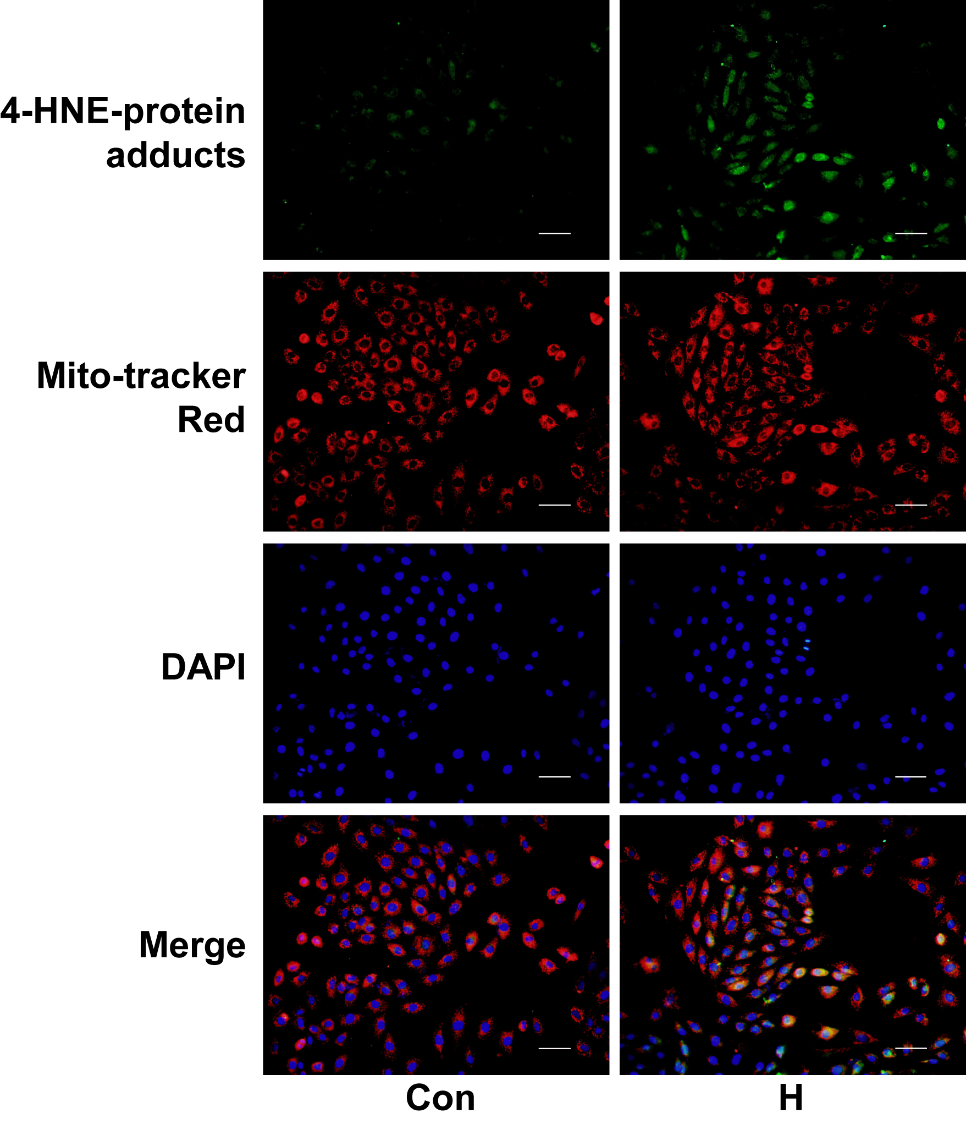
**

**Supplemental Figure 5.** The distribution of 4-HNE-protein adducts under hypoxia (H). Representative individual and merged fluorescence photographs of 4-HNE-protein adducts, Mito-tracker Red and DAPI staining. Scale bar = 250 μm.

# Supplementary Tables

**Supplemental Table 1. Baseline Characteristics of Rats in the ALDH2 Activation Study.**

|  | **Protocol 1** | | | **Protocol 2** | | |
| --- | --- | --- | --- | --- | --- | --- |
|  | **CA-CPR (*n* = 23)** | **CA-CPR+Alda-1 (*n* = 23)** | ***P*** | **CA-CPR (*n* = 12)** | **CA-CPR+Alda-1 (*n* = 12)** | ***P*** |
| Body weight (g) | 421.8 ± 10.5 | 434.6 ± 9.9 | 0.38 | 374.8 ± 10.0 | 364.3 ± 8.8 | 0.44 |
| Heart rate (beats/minute) | 413.2 ± 10.3 | 394.0 ± 12.4 | 0.24 | 448.8 ± 12.3 | 453.3 ± 9.2 | 0.77 |
| Mean arterial pressure (mm Hg) | 116.9 ± 4.5 | 116.9 ± 4.7 | 0.99 | 113.1 ± 4.5 | 114.5 ± 3.8 | 0.81 |
| Cardiac output (mL/minute)* | 31.8 ± 7.3 | 31.4 ± 7.1 | 0.96 | NA | NA | NA |
| Echocardiography† |  |  |  |  |  |  |
| Ejection fraction (%) | 82.3 ± 3.3 | 82.6 ± 1.5 | 0.95 | 84.6 ± 3.0 | 85.8 ± 2.6 | 0.76 |
| Fractional shortening (%) | 56.5 ± 1.1 | 57.6 ± 1.9 | 0.63 | 58.7 ± 2.1 | 56.2 ± 3.4 | 0.54 |
| LVEDV (μL) | 210.2 ± 21.4 | 200.2 ± 12.5 | 0.70 | 207.7 ± 42.4 | 229.8 ± 22.7 | 0.66 |
| LVESV (μL) | 30.9 ± 12.0 | 26.2 ± 3.8 | 0.72 | 34.6 ± 12.1 | 33.1 ± 8.1 | 0.92 |

Data are presented as mean ± SEM. **n* = 6 animals per group; †*n* = 5 animals per group. NA, not available.

**Supplemental Table 2. Procedural Indicators of Rats in the ALDH2 Activation Study.**

|  | **Protocol 1** | | | **Protocol 2** | | |
| --- | --- | --- | --- | --- | --- | --- |
|  | **CA-CPR (*n* = 23)** | **CA-CPR+Alda-1**  **(*n* = 23)** | ***P*** | **CA-CPR**  **(*n* = 12)** | **CA-CPR+Alda-1 (*n* = 12)** | ***P*** |
| Cardiac arrest duration (s)* | 271.3 ± 9.4 | 268.8 ± 10.4 | 0.86 | 234.7 ± 21.1 | 244.4 ± 9.7 | 0.68 |
| CPR duration (s)† | 84.6 ± 13.0 | 75.4 ± 12.7 | 0.61 | 67.5 ± 16.3 | 56.1 ± 14.5 | 0.62 |
| ROSC | 20 (87.0%) | 21 (91.3%) | 0.64 | 11 (91.7%) | 9 (75.0%) | 0.27 |

Data are presented as mean ± SEM or n (%). *The time from mean arterial pressure < 30 mm Hg to the start of CPR; †the time from the start of CPR to mean arterial pressure ≥ 60 mm Hg.

**Supplemental Table 3. Baseline Characteristics of Rats in the Cardiac Overexpression of ALDH2 Study.**

|  | **AAV9-Veh+**  **CA-CPR (*n* = 12)** | **AAV9-ALDH2+**  **CA-CPR (*n* = 12)** | ***P*** |
| --- | --- | --- | --- |
| Body weight (g) | 345.9 ± 8.9 | 345.6 ± 8.7 | 0.98 |
| Heart rate (beats/minute) | 392.1 ± 26.8 | 396.3 ± 22.5 | 0.91 |
| Mean arterial pressure (mm Hg) | 109.0 ± 7.3 | 118.7 ± 6.2 | 0.32 |
| Echocardiography* |  |  |  |
| Ejection fraction (%) | 89.8 ± 0.8 | 88.4 ± 1.1 | 0.34 |
| Fractional shortening (%) | 61.6 ± 1.9 | 58.0 ± 1.3 | 0.16 |
| LVEDV (μL) | 155.0 ± 10.9 | 134.5 ± 15.5 | 0.31 |
| LVESV (μL) | 16.1 ± 2.4 | 15.2 ± 1.4 | 0.75 |

Data are presented as mean ± SEM. *n = 5 animals per group.

**Supplemental Table 4. Procedural Indicators of Rats in the Cardiac Overexpression of ALDH2 Study.**

|  | **AAV9-Veh+CA-CPR (*n* = 12)** | **AAV9-ALDH2+CA-CPR (*n* = 12)** | ***P*** |
| --- | --- | --- | --- |
| Cardiac arrest duration (s)* | 258.3 ± 23.3 | 250.2 ± 29.7 | 0.83 |
| CPR duration (s)† | 98.6 ± 35.3 | 91.5 ± 36.6 | 0.89 |
| ROSC | 11 (91.7%) | 10 (83.3%) | 0.54 |

Data are presented as mean ± SEM or n (%). *The time from mean arterial pressure < 30 mm Hg to the start of CPR; †the time from the start of CPR to mean arterial pressure ≥ 60 mm Hg.

**Supplemental Table 5. Blood Gas After ROSC in Protocol 1 of the ALDH2 Activation Study.**

|  | **CA-CPR (*n* = 6)** | **CA-CPR+Alda-1 (*n* = 6)** | ***P*** |
| --- | --- | --- | --- |
| pH |  |  |  |
| 15 minutes | 7.1 ± 0.03 | 7.1 ± 0.05 | 0.65 |
| 1 hour | 7.2 ± 0.08 | 7.3 ± 0.02 | 0.10 |
| 4 hours | 7.3 ± 0.06 | 7.3 ± 0.05 | 0.82 |
| PaO_2_ (mm Hg) |  |  |  |
| 15 minutes | 108.3 ± 14.1 | 78.0 ± 9.7 | 0.11 |
| 1 hour | 102.0 ± 14.5 | 77.5 ± 14.4 | 0.26 |
| 4 hours | 123.0 ± 14.5 | 94.8 ± 20.9 | 0.29 |
| PaCO_2_ (mm Hg) |  |  |  |
| 15 minutes | 35.2 ± 3.7 | 31.8 ± 3.1 | 0.50 |
| 1 hour | 52.3 ± 9.2 | 33.8 ± 3.6 | 0.09 |
| 4 hours | 39.5 ± 15.6 | 31.4 ± 9.1 | 0.68 |
| Glucose (mmol/L) |  |  |  |
| 15 minutes | 14.4 ± 1.6 | 13.4 ± 1.9 | 0.69 |
| 1 hour | 13.9 ± 1.8 | 13.0 ± 1.5 | 0.71 |
| 4 hours | 10.7 ± 2.8 | 7.6 ± 1.9 | 0.42 |
| Lactate (mmol/L) |  |  |  |
| 15 minutes | 8.3 ± 1.4 | 7.7 ± 1.1 | 0.76 |
| 1 hour | 5.2 ± 1.4 | 4.0 ± 0.4 | 0.45 |
| 4 hours | 4.8 ± 1.5 | 5.1 ± 1.2 | 0.85 |

Data are presented as mean ± SEM.

**Supplemental Table 6. Blood Gas After ROSC in the Cardiac Overexpression of ALDH2 Study.**

|  | **AAV9-Veh+CA-CPR (*n* = 8)** | **AAV9-ALDH2+CA-CPR (*n* = 7)** | ***P*** |
| --- | --- | --- | --- |
| pH |  |  |  |
| 15 minutes | 7.1 ± 0.09 | 7.2 ± 0.09 | 0.58 |
| 1 hour | 7.2 ± 0.08 | 7.3 ± 0.08 | 0.47 |
| 4 hours | 7.3 ± 0.06 | 7.3 ± 0.02 | 0.66 |
| PaO_2_ (mm Hg) |  |  |  |
| 15 minutes | 96.0 ± 5.4 | 114.1 ± 7.3 | 0.06 |
| 1 hour | 97.1 ± 9.0 | 103.3 ± 10.0 | 0.66 |
| 4 hours | 97.6 ± 12.3 | 97.7 ± 5.1 | 0.99 |
| PaCO_2_ (mm Hg) |  |  |  |
| 15 minutes | 44.0 ± 6.3 | 30.6 ± 4.5 | 0.11 |
| 1 hour | 46.1 ± 10.1 | 40.3 ± 7.4 | 0.65 |
| 4 hours | 42.0 ± 5.4 | 45.8 ± 2.0 | 0.55 |
| Glucose (mmol/L) |  |  |  |
| 15 minutes | 17.8 ± 1.6 | 14.1 ± 1.4 | 0.10 |
| 1 hour | 16.1 ± 1.4 | 15.2 ± 1.1 | 0.61 |
| 4 hours | 12.2 ± 1.9 | 10.4 ± 1.0 | 0.42 |
| Lactate (mmol/L) |  |  |  |
| 15 minutes | 7.9 ± 1.9 | 8.7 ± 1.4 | 0.75 |
| 1 hour | 5.3 ± 1.5 | 5.4 ± 1.3 | 0.98 |
| 4 hours | 3.2 ± 0.9 | 2.6 ± 0.6 | 0.58 |

Data are presented as mean ± SEM.
